# *Plasmodium* alveolins possess distinct but structurally and functionally related multi-repeat domains

**DOI:** 10.1007/s00436-014-4226-9

**Published:** 2014-12-05

**Authors:** Fatimah S. Al-Khattaf, Annie Z. Tremp, Johannes T. Dessens

**Affiliations:** 1 Pathogen Molecular Biology Department, Faculty of Infectious and Tropical Diseases, London School of Hygiene & Tropical Medicine, Keppel Street, London, WC1E 7HT UK; 2 Department of Infection Control, College of Medicine, King Saud University, Riyadh, Saudi Arabia

**Keywords:** Cytoskeleton, Intermediate filament, Articulin, Tandem repeats

## Abstract

The invasive and motile life stages of malaria parasites (merozoite, ookinete and sporozoite) possess a distinctive cortical structure termed the pellicle. The pellicle is characterised by a double-layered ‘inner membrane complex’ (IMC) located underneath the plasma membrane, which is supported by a cytoskeletal structure termed the subpellicular network (SPN). The SPN consists of intermediate filaments, whose major constituents include a family of proteins called alveolins. Here, we re-appraise the alveolins in the genus *Plasmodium* with respect to their repertoire, structure and interrelatedness. Amongst 13 family members identified, we distinguish two domain types that, albeit distinct at the primary structure level, are structurally related and contain tandem repeats with a consensus 12-amino acid periodicity. Analysis in *Plasmodium berghei* of the most divergent alveolin, *Pb*IMC1d, reveals a zoite-specific expression in ookinetes and a subcellular localisation in the pellicle, consistent with its predicted role as a SPN component. Knockout of *Pb*IMC1d gives rise to a wild-type phenotype with respect to ookinete morphogenesis, tensile strength, gliding motility and infectivity, presenting the first example of apparent functional redundancy amongst alveolin family members.

## Introduction

Malaria parasite transmission begins when gametocytaemic blood is ingested by a vector mosquito. This initiates rapid gametogenesis followed by fertilisation. Zygotes transform into motile ookinetes that cross the midgut wall of the insect and transform into oocysts (Meis and Ponnudurai 1987; Meis et al. 1989). An approximately 2-week period of growth and replication concludes in hundreds of motile sporozoites being released from each oocyst and invading the salivary glands. Blood feeding of the insect transmits the sporozoites to the vertebrate host, where they replicate to each produce thousands of merozoites. The motile merozoites are released into the bloodstream, where they infect red blood cells and either replicate to form more merozoites or differentiate into sexual stage male and female gametocytes to complete the life cycle.

The three zoite stages of *Plasmodium* species (i.e. ookinetes, sporozoites and merozoites) possess a characteristic peripheral cytoskeletal structure known as the pellicle. The pellicle is defined by a double-membrane structure termed the inner membrane complex (IMC) (Bannister et al. 2000; Morrissette and Sibley 2002; Santos et al. 2009). The IMC is equivalent to a system of flattened membranous sacs that underlie the plasma membrane, the so-called ‘alveoli’, which are a defining feature of unicellular microorganisms belonging to the phyla Apicomplexa, Ciliophora (ciliates) and Dinoflagellata (dinoflagellates) within the protist Alveolata superphylum. Tightly associated with the IMC on its cytoplasmic side lies a network of intermediate filaments termed the subpellicular network (SPN), which supports the pellicular membranes and provides mechanical strength to the cell (Mann and Beckers 2001). Members of an Apicomplexa-specific family of proteins, termed IMC1 proteins, were identified as building blocks of the SPN (Khater et al. 2004; Mann and Beckers 2001). Subsequently, structurally related proteins from ciliates and dinoflagellates were identified and added to this protein family renamed ‘alveolins’ (Gould et al. 2008). In the genus *Plasmodium*, the alveolin family members display differential expression between different zoite stages of the parasite. In the rodent malaria species *Plasmodium berghei*, it was shown that disruption of individual alveolin family members expressed in sporozoites (*Pb*IMC1a), in ookinetes (*Pb*IMC1b) or in both these zoites (*Pb*IMC1h) results in morphological abnormalities that are accompanied by reduced tensile strength of the zoite stages in which they are expressed (Khater et al. 2004; Tremp and Dessens 2011; Tremp et al. 2008; Volkmann et al. 2012). Besides their roles in morphogenesis and tensile strength, the *Plasmodium* alveolins are also involved in gliding motility, most likely through interactions with components of the glideosome that are situated within the pellicular cytoplasm (Khater et al. 2004; Tremp and Dessens 2011; Tremp et al. 2008; Volkmann et al. 2012). Apart from their expression throughout the *Plasmodium* life cycle, alveolins are essential for parasite development both in the vertebrate and insect hosts (Khater et al. 2004; Tremp et al. 2014; Tremp and Dessens 2011), which makes them potentially attractive targets for malaria treatment, prophylaxis and transmission control. For this reason, it is important to better understand their core architecture, as well as the underlying mechanisms for their assembly into the supramolecular structures that make up the cortical cytoskeleton of the zoite stages.

In this study, we carried out a critical re-evaluation of the *Plasmodium* alveolins with respect to their repertoire, structure and interrelatedness. Our analyses identify two distinct domain types that are structurally and functionally related without possessing significant homology at the primary structure level.

## Materials and methods

### Animal use

All laboratory animal work undergoes regular ethical review by the London School of Hygiene & Tropical Medicine and has been approved by the UK Home Office. Work was carried out in accordance with the UK Animals (Scientific Procedures) Act 1986 implementing European Directive 2010/63 for the protection of animals used for experimental purposes. Experiments were conducted in 6–8-week-old female CD1 mice, specific pathogen free and maintained in filter cages. Animal welfare was assessed daily, and animals were humanely killed upon reaching experimental or humane endpoints. Mice were infected with parasites suspended in RPMI or phosphate-buffered saline (PBS) by intraperitoneal injection or by infected mosquito bite on anaesthetised animals. Parasitaemia was monitored regularly by collecting of a small drop of blood from a superficial tail vein. Drugs were administered by intraperitoneal injection or where possible were supplied in drinking water. Parasitised blood was harvested by cardiac bleed under general anaesthesia without recovery.

### Parasite maintenance, transmission, culture and purification

*P. berghei* ANKA clone 234 parasites were maintained as cryopreserved stabilates or by mechanical blood passage and regular mosquito transmission. Ookinete cultures were set up overnight from gametocytaemic blood as previously described (Arai et al. 2001). After 20–24 h, ookinetes were purified via ice-cold 0.17 M ammonium chloride lysis and centrifugation at 800×*g* for 10 min, followed by PBS washes. Mosquito infection and transmission assays were as previously described using *Anopheles stephensi* (Dessens et al. 1999; Khater et al. 2004), and infected insects were maintained at 20 °C at approximately 70 % relative humidity.

### Gene targeting constructs

The entire *pbimc1d* coding sequence plus ca. 0.55 kb of upstream sequence was PCR amplified from genomic DNA with primers pDNR-IMC1d-F (ACGAAGTTATCAGTCGAGGTACCAGCCAAAATCACCGAAAAG) and pDNR-IMC1d-R (ATGAGGGCCCCTAAGCTTTCAGATATTAAAGGAGCATTATCAATG) and cloned into *Sal*I/*Hin*dIII-digested pDNR-EGFP by in-fusion cloning to give plasmid pDNR-IMC1d/GFP. The 3′ untranslated region of *pbimc1d* was amplified with primers pLP-IMC1d-F (ATATGCTAGAGCGGCCTAGTAAGTCTTTTGCATTTTATCAATGC) and pLP-IMC1d-R (CACCGCGGTGGCGGCCAAAATATGAAGAAATGACAAAACAGAAG) and the resulting ca. 0.62-kb fragment cloned into *Not*I-digested pLP-hDHFR by in-fusion cloning to give plasmid pLP-hDHFR/IMC1d. The *pbimc1d/gfp*-specific sequence from pDNR-IMC1d/GFP was transferred to pLP-hDHFR/IMC1d by Cre/*lox*P recombination to give the final construct pLP-IMC1d/GFP. This plasmid served as template in PCR-based site-directed mutagenesis using primers IMC1d-KO-F (AGCCAGTGATGAGTAAAGGAGAAGAACTTTTCAC) and IMC1d-KO-R (TTACTCATCACTGGCTTATAAAATGCATTTATT). The resulting PCR product was circularised using in-fusion to give plasmid pLP-IMC1d-KO.

### Generation and genotyping of genetically modified parasites

Parasite transfection, pyrimethamine selection and dilution cloning were performed as previously described (Waters et al. 1997). Prior to performing transfections, plasmid DNA was digested with *Kpn*I and *Sac*II to remove the vector backbone. Genomic DNA extraction was performed as previously described (Dessens et al. 1999). Integration into the *pbimc1d* locus was confirmed with primers IMC1d-5′F (TACCCGCATATTTATCATTG) and LAP-GFP-R (GTGCCCATTAACATCACC), and the absence of the wild-type allele was confirmed using primers IMC1d-5′F and IMC1d-3′R (GGTTACATGTATTTTTATTTCCGC).

### RT-PCR analysis

Reverse transcription PCR (RT-PCR) analysis was carried out as described (Claudianos et al. 2002) using primers IMC1d-ORF-F (TTGAAAATGGAGATGCTATTACAAG) and pDNR-IMC1d-R (for *pbimc1d*) and primers tub1-F (GAAGTAATAAGTATACATGTAGG) and tub1-R (ACACATCAATGACTTCTTTACC) (for *pbtubulin1*).

### Western blot analysis

Parasite samples were heated directly in SDS-PAGE loading buffer at 70 °C for 10 min. Proteins were fractionated by electrophoresis through NuPage 4–12 % Bis-Tris precast gels (Invitrogen) and transferred to PVDF membrane (Invitrogen) according to the manufacturer’s instructions. Membranes were blocked for non-specific binding in PBS supplemented with 0.1 % Tween 20 and 5 % skimmed milk for 1 h at room temperature. Goat polyclonal antibody to GFP conjugated to horseradish peroxidase (Abcam ab6663) diluted 1:5000 was applied to the membrane for 1 h at room temperature. After washing, signal was detected by chemiluminescence (Pierce ECL western blotting substrate) according to the manufacturer’s instructions.

### Tensile strength and viability assays

Unpurified ookinetes present in ookinete cultures were subjected to hypo-osmotic shock of 0.5× normal osmotic strength by adding an equal volume of water. After 5 min, normal osmotic conditions were restored by adding an appropriate amount of 10× PBS. Cell viability was scored by fluorescence microscopy in the presence of 5 mL/L propidium iodide and 1 % Hoechst 33258. Ookinetes whose nucleus stained positive for both propidium iodide and Hoechst were scored as non-viable, whereas ookinetes whose nucleus only stained positive for Hoechst were scored as viable.

### Assessment of ookinete shape and motility

Images of Giemsa-stained ookinetes were captured by microscopy and their length and width measured. The ookinete motility assay was performed as previously described (Moon et al. 2009). Ookinete cultures were added to an equal volume of Matrigel (BD Biosciences) on ice, mixed thoroughly, spotted onto a microscope slide and covered with a Vaseline-rimmed cover slip. The Matrigel was allowed to set at room temperature for at 30 min. Time-lapse videos (one frame every 10 s for 10 min) were taken on a Zeiss Axioplan II microscope. Movies were analysed with ImageJ using the Manual Tracking plugin (http://fiji.sc/wiki/index.php/Manual_Tracking).

### Microscopy

For assessment of fluorescence, live parasite samples were assessed and images captured on a Zeiss LSM510 inverted laser scanning confocal microscope.

### Bioinformatics

Conserved domains were identified by multiple alignments of orthologous proteins from *P. berghei*, *P. falciparum*, *P. vivax* and *P. knowlesi*. Multiple alignments were obtained using Clustal Omega, and phylogenetic analyses were carried out using ClustalW2 Phylogeny, accessed through the EMBL-EBI website. Amino acid sequence similarity searches were carried out by protein BLAST, accessed through the National Centre for Biotechnology Information (NCBI), PlasmoDB or ToxoDB. Trees were drawn using TreeDraw. The program HHrepID was accessed through the Bioinformatics Toolkit, Max-Planck Institute for Developmental Biology.

## Results

### Repertoire and interrelatedness of *Plasmodium* alveolins

The existence of an alveolin protein family in *Plasmodium* was first reported in 2004, identifying eight putative members named IMC1a through to IMC1h (Khater et al. 2004). More recent studies identified several additional alveolins (Gould et al. 2008; Kono et al. 2012; Tremp et al. 2013), here named IMC1i to IMC1l in keeping with original nomenclature (Table 1). To evaluate the structural interrelatedness of these alveolins, we carried out a systematic analysis of the *Plasmodium* genome using BLAST similarity searches with each of the family members. In the process, we identified a 13th family member, named IMC1m (Table 1). Alveolin hits from each of the BLAST searches were given an arbitrary integer score (relating to the scores of the BLAST hits, lowest score receives 1) to generate a similarity matrix (Table 2). For each alveolin, a total score was then calculated to reflect its structural similarity to the *Plasmodium* alveolin family as a whole. This, in turn, allowed a ranking of the 13 alveolins with respect to their interrelatedness. Accordingly, IMC1e (ranked 1) was identified as being structurally most similar to the alveolin population: it both detects and is detected by the highest number of family members (Table 2). In contrast, IMC1d (ranked 13) has the most divergent structure (Table 2). This suggests that IMC1e represents the most recent common ancestor. Indeed, *Pb*IMC1e was much more successful than *Pb*IMC1d at detecting alveolins in other genera within the Apicomplexa phylum. For example, in *Toxoplasma gondii*, which encodes 14 alveolins (named *Tg*IMC1 and *Tg*IMC3–*Tg*IMC15) (Anderson-White et al. 2011), *Pb*IMC1e detected 13 family members in protein BLAST, whilst *Pb*IMC1d detected five.

**Table 1:** Predicted *Plasmodium* IMC1 proteins/alveolins and zoite stage expression

Name	*P. berghei* gene ID (PBANKA_000000)	*P. falciparum* gene ID (PF3D7_0000000)	Alternative name(s)	Zoite expression			References
				Merozoite	Ookinete	Sporozoite	
IMC1a	040260	0304000	Alv1			+	Khater et al. 2004
IMC1b	090710	1141900			+		Tremp et al. 2008
IMC1c	120200	1003600	Alv5	+	+	+	Tremp et al. 2014
IMC1d	121910	0708600	hsp90, hsp86, o2		+		this paper
IMC1e	040270	0304100	Alv2	+	+	+	Tremp et al. 2014
IMC1f	136440	1351700	Alv6				
IMC1g	124060	0525800	Alv4	+	+	+	Kono et al. 2012
IMC1h	143660	1221400	Alv3		+	+	Tremp and Dessens 2011
IMC1i	070710	0823500					
IMC1j	112040	0621400	Alv7, Pfs77				
IMC1k	135490	1341800					
IMC1l	102570	1417000					
IMC1m	051300	1028900

**Table 2:** Similarity matrix and ranking of *Plasmodium berghei* alveolins

*Pb*IMC1	Hit^a^	a	b	c	d	e	f	g	h	i	j	k	l	m	
Query															
a			9	8	7	5	6	2	0	1	3	4	0	0	45
b		5		2	4	0	3	0	0	0	1	0	0	0	15
c		11	5		0	9	10	8	7	6	2	4	1	3	66
d		3	2	0		0	1	0	0	0	0	0	0	0	6
e		5	1	9	0		7	11	8	10	3	4	2	6	66
f		3	1	4	0	5		0	2	0	0	0	0	0	15
g		1	0	8	0	10	5		7	9	2	6	3	4	55
h		0	0	5	0	7	4	6		1	0	2	0	3	28
i		0	0	3	0	4	2	5	0		0	0	1	0	15
j		1	8	3	0	7	2	6	0	4		9	0	5	45
k		7	0	5	0	6	3	4	1	2	8		0	0	36
l		0	0	0	0	2	3	4	0	1	0	0		0	10
m		1	0	7	0	9	8	5	6	2	4	0	3		45
		37	26	54	11	64	54	51	31	36	23	29	10	21	Total^c^
Ranking^b^		4	11	2	13	1	5	3	9	10	6	8	12	7	

^a^Each ‘Query’ alveolin was run with protein-protein BLAST against the *P. berghei* genome in PlasmoDB. The lowest scoring ‘Hit’ alveolin was given an arbitrary similarity score of 1, the second lowest a score of 2, and so forth. A 0 value meant the alveolin was not detected

^b^Ranking was done by combining the bottom and right-hand side ‘Total’ scores for each alveolin. The highest scoring alveolin was ranked 1

^c^Total scores reflect the ability of each alveolin to detect (right-hand side), or to be detected by (bottom), other alveolins in the BLAST similarity search

### Domain structure of *Plasmodium* alveolins

**Fig. 1:**
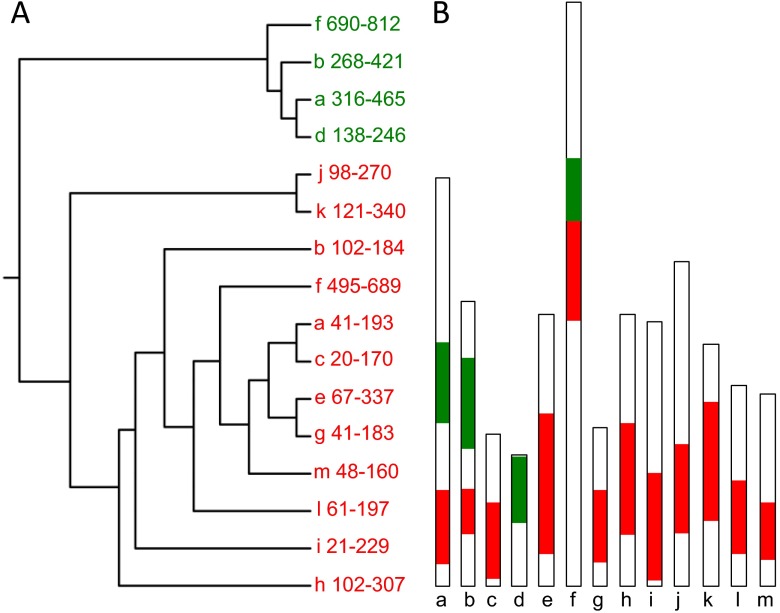
Repertoire and domain structure of *Plasmodium* alveolins. **a** Phylogeny of conserved domains within the alveolin family members *Pb*IMC1a to *Pb*IMC1m (*a–m*). *Numbers* give amino acid coordinates of the conserved domains in the corresponding *Pb*IMC1 protein. Type 1 (*red*) and type 2 (*green*) domains separate into distinct clades. **b** Schematic diagram depicting the 13 alveolin family members (*a–m*), showing relative positions of the type 1 (*red*) and type 2 (*green*) domains

*Plasmodium* alveolins are typified by possessing, within their primary amino acid sequences, highly conserved regions that are flanked by sequences more variable in length and amino acid composition (Khater et al. 2004; Tremp et al. 2008, 2014; Tremp and Dessens 2011). The functional properties of the alveolins are likely to be defined by these conserved domains. Closer examination showed that the sequence similarities between the alveolins identified from the BLAST searches (Table 2) were largely confined to two conserved domains, here named type 1 and type 2, which by phylogenetic analysis split into distinct clades and which are variably distributed amongst the family members (Fig. 1). The alveolins *Pb*IMC1a and *Pb*IMC1b are the only family members that possess interspersed type 1 and type 2 domains (Fig. 1b) (Khater et al. 2004; Tremp et al. 2008). Whilst these different domain types share little sequence homology, both have a strong compositional bias for the amino acids P, I, V, D, E and K (e.g. 66 % for *Pb*IMC1a type 1, 62 % for *Pb*IMC1a type 2, 64 % for *Pb*IMC1b type 1, 66 % for *Pb*IMC1b type 2). These observations suggested that the type 1 and type 2 domains could be structurally related, despite a lack of discernible primary amino acid sequence homology.

### *Pb*IMC1d is expressed in ookinetes and localises to the pellicle/SPN

*Pb*IMC1d is not only the most divergent alveolin family member (Table 2) but is the only alveolin that possesses only a type 2 domain (Fig. 1). We used *Pb*IMC1d to assess the functional relationship between the type 1 and type 2 domains by determining its life stage expression, subcellular distribution and contribution to parasite development. *Pb*IMC1d is encoded by a two-exon gene, separated by a 170-bp intron. The gene is annotated as a putative heat shock protein 90 (hsp90) in *P. berghei* (hsp86 in *P. falciparum*), but it has no actual sequence similarity to heat shock proteins. The full-length protein is composed of 249 amino acids with a calculated *M*_*r*_ of 29,361. The type 2 domain in *Pb*IMC1d is highly conserved and has a 68 % amino acid content composed of P, I, V, D, E and K.

**Fig. 2:**
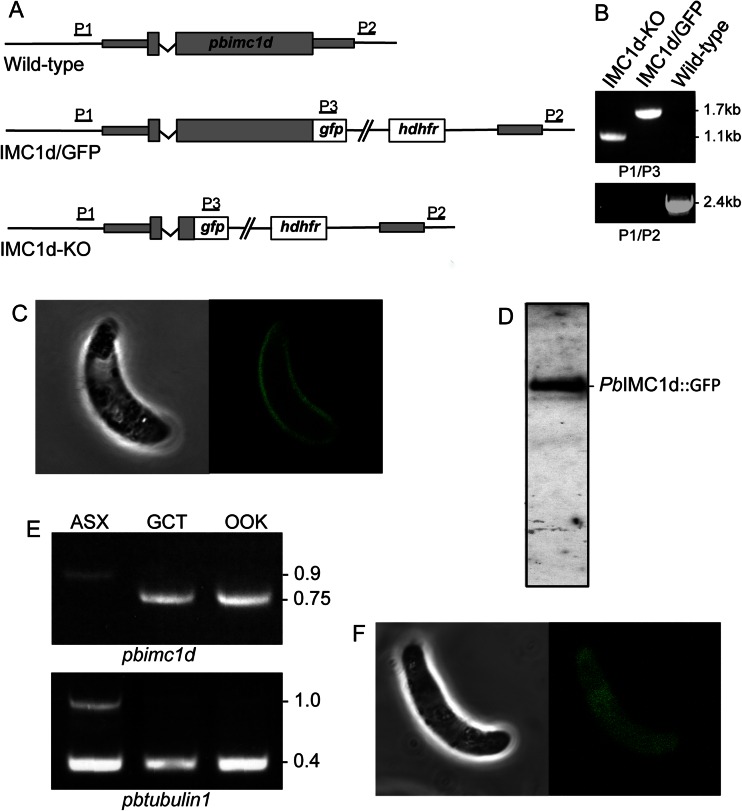
Generation and molecular analyses of genetically modified parasite lines. **a** General targeting strategy for the GFP tagging and gene disruption of *pbimc1d* via double crossover homologous recombination. Both wild-type (WT) GFP-tagged (IMC1d/GFP) and disrupted (IMC1d-KO) alleles are shown. The *pbimc1d* gene is indicated with coding sequence (*wide bars*) and non-coding sequence (*narrow bars*). Also indicated are the enhanced GFP module (*gfp*), the hDHFR selectable marker gene cassette (*hdhfr*) and primers used for diagnostic PCR amplification (*P1–P3*). **b** PCR diagnostic for the presence of the modified GFP-tagged *pbimc1d* alleles using primers P2 and P3 and the absence of the wild-type *pbimc1d* allele using primers P1 and P2, from clonal parasite populations of IMC1d/GFP and IMC1d-KO. WT parasites are included as positive controls for the wild-type alleles. **c** Confocal brightfield and GFP fluorescence image of a cultured, mature ookinete of parasite line IMC1d/GFP, showing cortical fluorescence. **d** Western blot analysis of purified, cultured ookinetes of parasite lines IMC1d/GFP using anti-GFP antibodies, showing the *Pb*IMC1d::GFP fusion protein. **e** RT-PCR analysis of wild-type parasite samples enriched for asexual stages (*ASX*), gametocytes (*GCT*) and ookinetes (*OOK*) using primers specific for *pbimc1d* and *pbtubulin1*. Due to the primers flanking introns, for each gene, the larger PCR products are amplified from gDNA and the smaller from cDNA. **f** Confocal brightfield and GFP fluorescence image of a cultured, mature ookinete of parasite line IMC1d-KO, showing cytoplasmic fluorescence

*Pb*IMC1d expression and localisation was studied by tagging the gene with enhanced green fluorescent protein (GFP) in genetically modified parasites. To achieve this, we used a strategy of double crossover homologous recombination in which the wild-type allele was replaced with a recombinant full-length wild-type allele fused to enhanced GFP at its carboxy terminus (Fig. 2a), giving rise to stably transfected parasites. To study the function of *Pb*IMC1d, we generated a null mutant using a similar gene targeting strategy, but removing most of the *pbimc1d* coding sequence whilst leaving the *gfp* gene under control of the native *pbimc1d* promoter to act as a reporter (Fig. 2a). After transfection of purified schizonts, pyrimethamine-resistant parasites were selected and cloned by limiting dilution as described (Tremp and Dessens 2011; Tremp et al. 2008) to give parasite lines IMC1d/GFP and IMC1d-KO, respectively. PCR diagnostic for integration into the *pbimc1d* locus produced a specific band of approximately 1.1 kb in the IMC1d-KO parasite, whilst this product was approximately 1.7 kb in parasite line IMC1d/GFP (Fig. 2b). The size difference between these PCR products reflects the removal of the *pbimc1d* coding sequence in the null mutant. PCR diagnostic for the presence of the wild-type *pbimc1d* allele gave a specific band of approximately 2.4 kb only in wild-type parasites (Fig. 2b). Parasite line IMC1d/GFP developed normally in mouse and mosquito. GFP fluorescence was only observed in zygotes and ookinetes and in the latter was predominantly distributed at the cell cortex (Fig. 2c). Immunoblot analysis of purified, cultured ookinetes with anti-GFP antibodies detected a specific band corresponding to the *Pb*IMC1d::GFP fusion protein (Fig. 2d). These data show that *Pb*IMC1d is specifically expressed in only one of the zoite stages, the ookinete, and is targeted to the pellicle structure/SPN. In *T. gondii*, the ‘alveolin domain’ was shown to be the main determinant in targeting the protein to the cell cortex (Anderson-White et al. 2011). Thus, the pellicular localisation of *Pb*IMC1d in ookinetes suggests that the type 2 domain in its own right is able to target proteins to this cellular compartment, as does the type 1 domain in other *Plasmodium* alveolins (Tremp et al. 2014; Tremp and Dessens 2011).

Expression profiling of *pbimc1d* messenger RNA by RT-PCR with *pbimc1d*-specific primers flanking its intron amplified an approximately 0.75-kb messenger RNA (mRNA)-specific product in both gametocytes and ookinetes, but in asexual blood stages only amplified an approximately 0.9-kb product from genomic DNA (this product is larger in size due to the intron) (Fig. 2e). By contrast, mRNA of the control gene tubulin1 was present in all parasite samples (Fig. 2e). The discrepancy between *pbimc1d* mRNA and *Pb*IMC1d protein expression in gametocytes strongly points to translational repression of the *pbimc1d* gene, as is predicted for the majority of alveolins in *P. berghei* (Mair et al. 2006). Translational repression is a female gametocyte-specific mechanism of translational silencing involved in the development of the parasite post-fertilisation (Mair et al. 2006).

### *Pb*IMC1d is functionally redundant

Ookinetes of the *Pb*IMC1d-null mutant displayed cytoplasmic GFP fluorescence resulting from expression of the GFP reporter from the *pbimc1d* promoter (Fig. 2f), which is consistent with the expression pattern of *Pb*IMC1d as determined by GFP tagging. The cell shape of *Pb*IMC1d-null mutant ookinetes was not significantly different from that of control IMC1d/GFP ookinetes (length 11.49 ± 0.10 μm for IMC1d/GFP, 11.26 ± 0.12 μm for IMC1d-KO; width 2.15 ± 0.036 μm for IMC1d/GFP, 2.23 ± 0.034 μm for IMC1d-KO; *n* = 100), and the ookinetes were equally effective in producing oocysts in mosquitoes (140 ± 39 oocysts per mosquito for IMC1d/GFP, 140 ± 28 for IMC1d-KO; *n* = 20), indicating that they possess normal tensile strength and motility. Indeed, ookinetes of both parasite lines displayed similar resistance to hypo-osmotic shock (78 and 76 % survival for IMC1d/GFP and IMC1d-KO, respectively; *n* = 100), indicating that the knockout did not adversely affect tensile strength. Furthermore, ookinete motility through Matrigel was comparable between the two parasite lines (42.6 ± 1.7 μm per 10 min for IMC1d/GFP; 41.3 ± 2.6 μm for IMC1d-KO; *n* = 15). Finally, sporozoites of both parasite lines were readily transmitted by mosquito bite. Thus, *Pb*IMC1d appears to be functionally redundant under our experimental conditions.

### Alveolins possess tandem repeat sequences with a 12-amino acid periodicity

**Fig. 3:**
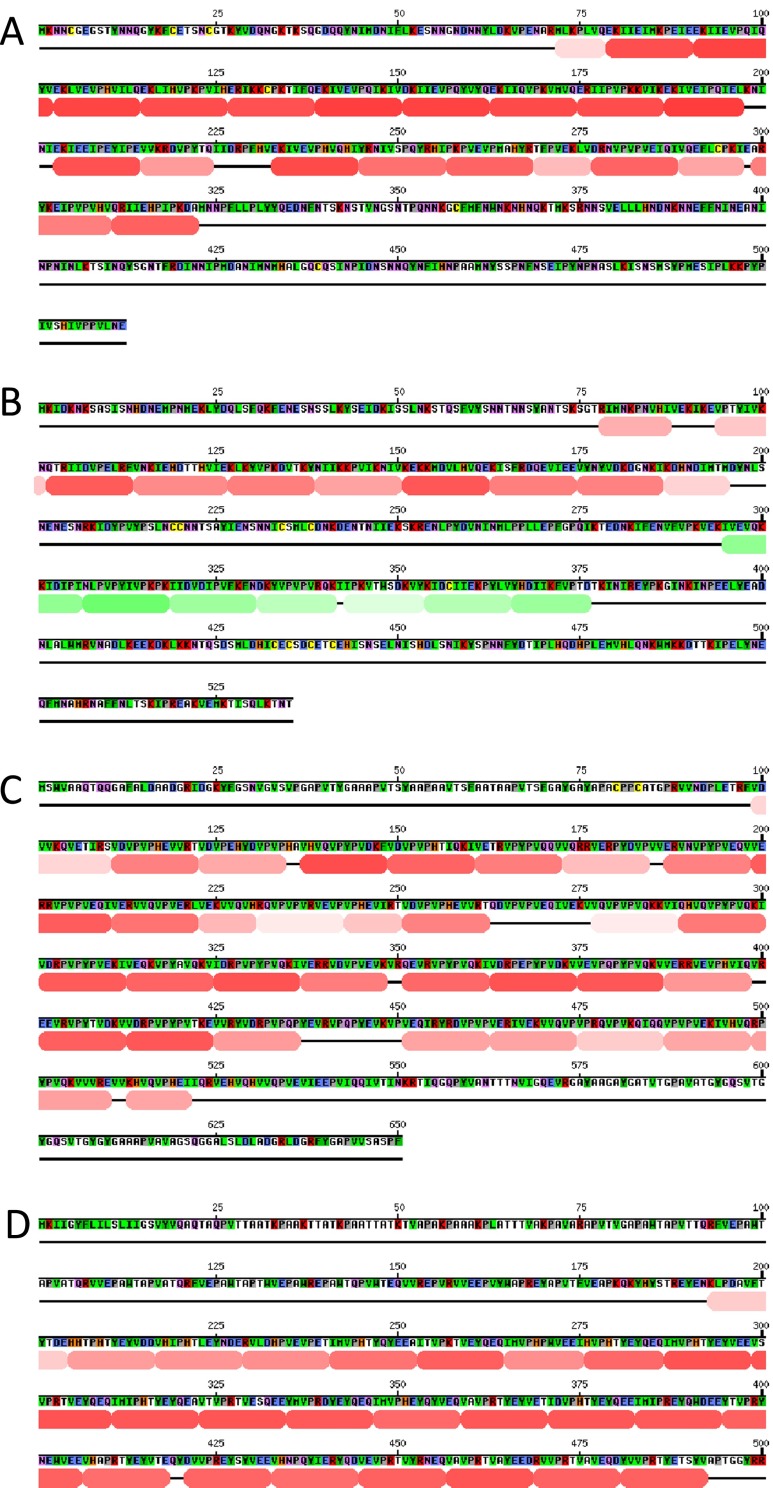
Tandem repeat identification in alveolins, articulins and plateins by the program HHrepID. **a** *P. berghei* IMC1e. **b** *P. berghei* IMC1b. **c** *Euglena gracilis* articulin (AAB23241.1). **d** *Euplotes aediculatus* alpha-2 platein precursor (AAM94463.1)

Alveolins have been reported to possess a core of repeated sequence motifs (Gould et al. 2008). We tested each of the family members for the presence of tandem repeat sequences using the program HHrepID. This method predicts structural repeats in protein sequences based on Hidden Markov Model (HMM)-HMM comparison, exploiting evolutionary information derived from multiple sequence alignment of homologues (Biegert and Soding 2008). These analyses revealed that all 13 *Plasmodium* alveolins possess predicted multi-repeat sequences within their conserved type 1 and type 2 domains, supporting the notion that they constitute genuine alveolins. Repeats within the large majority of type 1 domains revealed a clear minimum periodicity of 12 residues (e.g. *Pb*IMC1e, Fig. 3a). Tandem 12-amino acid repeats were also identified in type 2 domains (e.g. *Pb*IMC1b, Fig. 3b), albeit typically with lower probability scores. Articulins and plateins, cortical cytoskeleton proteins from *Euglena* and *Euplotes* spp., respectively, have also been reported to have 12-amino acid repetitive motifs rich in valine and proline residues and, in this respect, are similar to alveolins (Huttenlauch et al. 1995; Huttenlauch and Stick 2003; Kloetzel et al. 2003a, b; Marrs and Bouck 1992). This was confirmed by HHrepID analysis, which readily detected 12-amino acid tandem repeat structures in these proteins (Fig. 3c, d).

## Discussion

This study brings the number of *Plasmodium* alveolin family members that have been identified to 13. Of these, seven have now experimentally been shown to localise to the pellicle structure. This includes the most divergent family member, IMC1d, characterised here, which gives strong support to the concept that all *Plasmodium* alveolins are fundamentally involved with the cortical cytoskeleton in malaria parasites. This is supported by several alveolin knockout studies that reveal key roles in morphogenesis, tensile strength and gliding motility. Studies involving double knockout of *Pb*IMC1b and *Pb*IMC1h showed that the functional contribution of these alveolins to tensile strength and motility is both cumulative and mutually independent (Tremp and Dessens 2011). In contrast, the apparent no-phenotype knockout of *Pb*IMC1d reported here points to a functional redundancy amongst the alveolin family members. The highly conserved type 2 domain of IMC1d indicates that the gene is under selective pressure, and we cannot rule out that the phenotype of the *Pb*IMC1d-null mutant is very subtle, or might only become apparent under conditions that are different from our experimental set-up. However, another possible explanation for the seemingly redundant role of *Pb*IMC1d is that the formation and function of the SPN entail a system of co-operating proteins that are able to bypass, or substitute for, each other’s functions. This attractive hypothesis, if true, would add robustness to a complex biological system that serves roles in many key processes such as morphogenesis, motility and invasion.

The creation of internal repetitions forms an important mechanism for proteins to adapt their structure and function under evolutionary pressure (Marcotte et al. 1999). However, after fixation of duplications, sequence similarities amongst repeats can quickly erode whilst structure and function are preserved (Andrade et al. 2001). Although such mismatch or ‘fuzzy’ repeats are widespread, they are difficult to detect due to their low similarity, polymorphism and vast diversity. This is further confounded by the potential for non-canonical repeats, degenerate repeats and discontinuities (i.e. short insertions between repeats) to arise, which for example are known to frequently occur in coiled-coil domains, a well-known type of tandem repeat structure with a consensus 7-amino acid periodicity (Brown et al. 1996). The type 1 and type 2 domains identified here in the *Plasmodium* alveolins are likely to be the result of similar evolutionary forces. These alveolin domains lack discernible homology at the primary structure level and fall into distinct clades, but are clearly related with regard to their amino acid composition, tandem repeat structure and functional localisation. There is further indication that the type 2 repeats arose from type 1 repeats (or vice versa). First, in IMC1f, the type 1 and type 2 domains are located tandemly within a single uninterrupted block of conserved sequence (Fig. 1b). Second, four *T. gondii* alveolins share sequence similarity in protein BLAST with both *Pb*IMC1e and *Pb*IMC1d. In three of these (*Tg*IMC6, *Tg*IMC14 and *Tg*IMC15), the regions of sequence similarity with *Pb*IMC1e and *Pb*IMC1d overlap, pointing to the presence of domains with ‘intermediate’ homology between type 1 and type 2. These combined observations suggest that the primary amino acid sequences of the type 1 and type 2 repeats have evolved in a way that has preserved their overall structure and function. This phenomenon of ‘constrained evolution’ has also been observed in other protist cytoskeletal proteins with repeat motifs (Gould et al. 2011).

Many bioinformatics programs for internal repeat detection in proteins utilise algorithms based on local alignment and substitution matrices (Andrade et al. 2000; Heger and Holm 2000). These approaches have been modestly successful in predicting tandem repeats within the alveolins. By contrast, HHrepID is based on building and matching Hidden Markov Models to identify repeat sequences (Biegert and Soding 2008). Our findings demonstrate that HHrepID successfully predicts tandem repeats in the *Plasmodium* alveolins and show for the first time that these repeats have a consensus 12-amino acid length. Accordingly, the multi-repeat structures of alveolins are very similar, both in periodicity and amino acid composition, to those of cytoskeletal proteins in other protists: the articulins and plateins. Whilst *Euplotes* plateins possess canonical ER signal peptides and form plate-like structures inside alveoli (Kloetzel et al. 2003b), *Euglena* articulins form intermediate filaments with a more classic membrane skeleton role (Huttenlauch et al. 1995). Our findings give support to the concept that articulins, plateins and alveolins have a common structural scaffold and thus should be grouped within the same protein superfamily. Structural studies are being carried out to test this hypothesis. Interestingly, prokaryotic articulin homologues have been discovered in *Caulobacter crescentus*, the first bacterium described to rely on an intermediate filament-based cytoskeleton for its cell shape (Ausmees et al. 2003). Thus, articulin-like proteins like alveolins could be far more widespread than originally assumed.
